# Monopolar versus bipolar transurethral resection of bladder Tumour: post-hoc analysis of a prospective trial

**DOI:** 10.1007/s00345-024-05124-9

**Published:** 2024-08-02

**Authors:** Chris Ho-Ming Wong, Joycelyn Yung-Yung Lim, Ivan Ching-Ho Ko, David Ka-Wai Leung, Steffi Kar-Kei Yuen, Siu-Ying Yip, Chi-Fai Ng, Jeremy Yuen-Chun Teoh, Eddie Shu-Yin Chan

**Affiliations:** 1 S.H. Ho Urology Centre, Department of Surgery, Faculty of Medicine, The Chinese University of Hong Kong, Hong Kong SAR, China; 2 Department of Urology, Medical University of Vienna, Vienna, Austria; 3 Department of Surgery, Prince of Wales Hospital, 4/F, Clinical Sciences Building, New Territories, Hong Kong SAR China

**Keywords:** Transurethral resection of bladder tumour (TURBT), Monopolar TURBT, Bipolar TURBT, Resection quality, Oncological outcomes, Non-muscle invasive bladder cancer, NMIBC

## Abstract

**Introduction:**

Previously, in a randomised trial we demonstrated bipolar transurethral resection of bladder tumor (TURBT) could achieve a higher detrusor sampling rate than monopolar TURBT. We hereby report the long-term oncological outcomes following study intervention.

**Methods:**

This is a post-hoc analysis of a randomized phase III trial comparing monopolar and bipolar TURBT. Only patients with pathology of non-muscle invasive bladder cancer (NMIBC) were included in the analysis. Per-patient analysis was performed. Primary outcome was recurrence-free survival (RFS). Secondary outcomes included progression-free survival (PFS), cancer-specific survival (CSS) and overall survival (OS).

**Results:**

From the initial trial, 160 cases were randomised to receive monopolar or bipolar TURBT. 24 cases of non-urothelial carcinoma, 22 cases of muscle-invasive bladder cancer, and 9 cases of recurrences were excluded. A total of 97 patients were included in the analysis, with 46 in the monopolar and 51 in the bipolar group. The median follow-up was 97.1 months. Loss-to-follow-up rate was 7.2%. Regarding the primary outcome of RFS, there was no significant difference (HR = 0.731; 95%CI = 0.433–1.236; *P* = 0.242) between the two groups. PFS (HR = 1.014; 95%CI = 0.511–2.012; *P* = 0.969), CSS (HR = 0.718; 95%CI = 0.219–2.352; *P* = 0.584) and OS (HR = 1.135; 95%CI = 0.564–2.283; *P* = 0.722) were also similar between the two groups. Multifocal tumours were the only factor that was associated with worse RFS.

**Conclusion:**

Despite the superiority in detrusor sampling rate, bipolar TURBT was unable to confer long-term oncological benefits over monopolar TURBT.

**Supplementary Information:**

The online version contains supplementary material available at 10.1007/s00345-024-05124-9.

## Introduction

Bladder cancer is the 10th most common cancer worldwide, with an estimated 573,000 new cases and 213,000 deaths in 2020 [1, 2]. Approximately 75% of newly diagnosed bladder cancers are non-muscle invasive bladder cancer (NMIBC) [3]. Transurethral resection of bladder tumor (TURBT) is the gold standard treatment for NMIBC, providing essential diagnostic and therapeutic roles [4, 5].

Both monopolar and bipolar TURBT techniques have been available for the surgical management of NMIBC for years. Historically monopolar TURBT had been the mainstay of treatment for decades, but it was associated with potential concerns such as obturator nerve reflex, bladder perforation, and thermal damage to the bladder wall [6]. Bipolar TURBT on the other hand has offered potential advantages such as a more precise resection, reduced tissue charring, improved haemostasis, and the ability to use isotonic saline as an irrigation fluid, minimizing the potential risk of TUR syndrome [7].

Several studies have compared monopolar and bipolar TURBT in terms of perioperative outcomes and complications. A meta-analysis by Cui et al. found that bipolar TURBT was associated with shorter operative time, lower transfusion rates, and reduced bladder irrigation and catheterization times compared to monopolar TURBT [8]. However, despite these potential advantages, there remains a lack of consensus regarding the superiority of bipolar TURBT over monopolar TURBT, particularly in terms of long-term oncological outcomes.

The quality of TURBT, as determined by factors such as completeness of resection and presence of detrusor muscle in the specimen, has been shown to impact recurrence and progression rates in NMIBC [9]. We have reported the early results of this randomised controlled trial before [10]. The primary outcome of the study was detrusor muscle sampling rate from monopolar versus bipolar TURBT. In the study, we discovered that there was a trend towards a better sampling rate in the bipolar arm. A difference of 17% of detrusor muscle sampling rate was reported. However, whether these potential advantages translate into improved long-term oncological outcomes remains uncertain. Therefore, we would like to report the long-term results with a focus on the oncological outcomes from the trial. Given the paucity of data on the comparative oncological efficacy of monopolar and bipolar TURBT, we believe the results will add value to the available literatures.

## Methods and materials

### Study design

The current analysis was based on data yielded from a single centre phase III randomised controlled trial (RCT) conducted in a tertiary academic centre [10]. Post-hoc analysis was attempted to identify the oncological outcomes of the monopolar and bipolar TURBT arms.

The details of the protocol of the trial were previously published and reported [10]. For additional information *including the criteria of patient selection; randomization*,* allocation concealment*,* and blinding*,* treatment protocol*,* and sample size calculation*, please refer to supplementary information (1) for details.

### Ethics approval

The study was registered with ClinicalTrials.gov (NCT01581723). Prior to the commencement of the study, ethics approval from the local authority had been granted. It was conducted in accordance with good clinical practice and the Declaration of Helsinki.

### Study outcomes and post-hoc analysis

The aim of the current study is to identify the long-term oncological outcomes of monopolar versus bipolar TURBT for NMIBC. In this analysis, only patients with final pathology of NMIBC were included. Patients with benign pathology or muscle-invasive bladder cancer were excluded. Primary outcome was recurrence-free survival (RFS). Bladder cancer recurrence was based on endoscopic and histological diagnosis. Secondary outcomes included progression-free survival (PFS), cancer-specific survival (CSS) and overall survival (OS). Other outcomes that would be reported in the analysis included the peri-operative complication profiles (graded according to the Clavien-dindo classification).

### Data collection and follow-up

Baseline patient characteristics were obtained via the electronic documentation system. Data were collected prior to the admission for operation. Intraoperative details and perioperative outcomes were documented on a pre-set electronic form, with the aid of accessing patient charts. Patients would attend follow-up 4 weeks ± 2 weeks after the operation for pathology review and assessment of early postoperative outcomes. They had subsequent follow-up every 3 months since surgery until 12 months. Check cystoscopy was performed at every visit. Subsequent interval of check cystoscopy was based on EAU guidelines [11]. Low risk cases would receive cystoscopy every year. For intermediate risk patients, subsequent cystoscopy would be arranged every 6 months. High risk patients would be receiving cystoscopy every 3 months till two years post operation, then every 6 months for five years. Additional investigations such as computed tomography, positron emission tomography scan or magnetic resonance imaging would be arranged according to physician judgement.

### Statistical analysis

Statistical analyses were performed with SPSS version 24.0 (IBM Corporation, Armonk, NY, USA). Analysis was performed after applying the inclusion and exclusion criteria to the original cohorts. Categorical variables would be presented as percentages, and continuous variables would be presented as mean with standard deviation, or median with interquartile ranges. Independent sample t-test was used for parametric continuous variables and the chi-square test was used for categorical variables. A p-value of < 0.05 was statistically significant. Kaplan-Meier survival plots would be adopted for the identification of the primary outcomes. Multivariate Cox regression analysis would be performed to identify contributing factors for RFS and PFS.

## Results

**Fig. 1:**
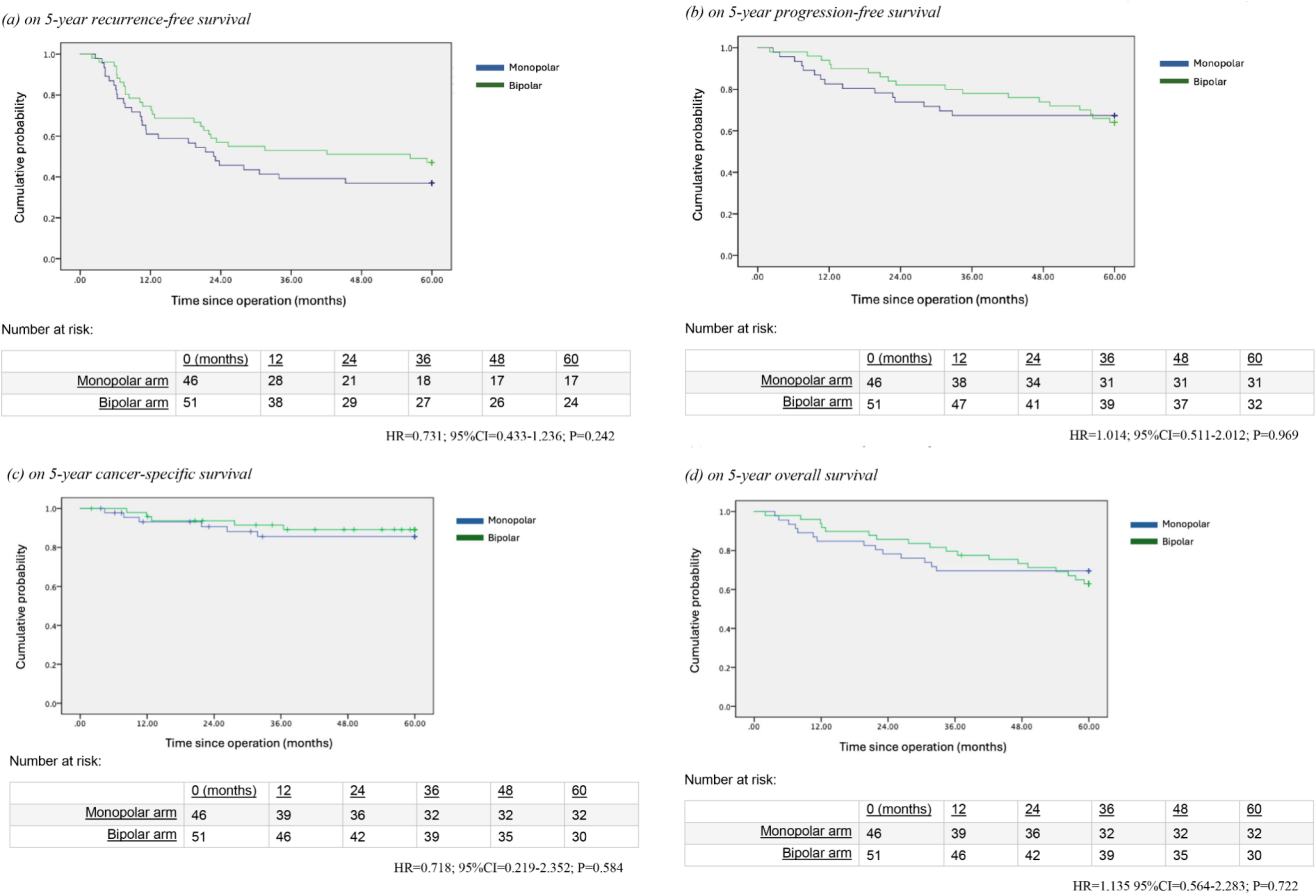
Kaplan-Meier analysis of survival outcomes

The Consolidated Standards of Reporting Trials (CONSORT) [12] diagram in the supplementary information (2). From May 2012 to December 2015, 335 patients were assessed for study eligibility. 40 patients had received a previous TURBT within a 6-week period and 135 patients declined to participate in the study, so they were not involved. 160 patients were recruited and analysed. 80 patients were allocated to each of the monopolar TURBT and bipolar TURBT groups. All the patients in the monopolar group received the intended intervention. One patient in the bipolar group did not receive the intended intervention as he was medically unfit. After adopting the exclusion criteria, a total of 97 patients were analysed. In the monopolar group, 34 patients were excluded from analysis while there were 28 patients in the bipolar group excluded. The reasons of exclusion were listed in Fig. 1. In the monopolar arm, 14 patients had a non-urothelial carcinoma pathology, 11 patients were diagnosed as MIBC and 5 counts of TURBT were performed on repeated patients. In the bipolar arm, 10 cases were not urothelial carcinoma, 11 cases were MIBC, and the 4 counts of operations were repeated instances.

In the final analysis, the monopolar group consisted of 46 patients and the bipolar group had 51 patients. The median follow-up duration is 97.1 months in the monopolar arm and 96.8 months in the bipolar arm. 4 patients in the monopolar group and 3 patients in the bipolar group were lost to follow up.

Table 1 shows the baseline characteristics of the cases in the two arms. Patient and tumour characteristics were similar across the groups. 71.7% of the cases in the monopolar group were male, and 78.4% in the bipolar group were male. Single tumour cases accounted for 54.3% of the monopolar group and 56.9% of the bipolar group. 58.7% of the cases in the monopolar group were Ta stage whereas the figure was 74.5% in the bipolar group (*p* = 0.1). Tumour grade was also comparable, with 89.1% of the cases in the monopolar group and 78.4% in the bipolar group being Grade 2 or above. During follow-up, 1 patient in the monopolar arm and 3 patients in the bipolar arm had received intravesical Bacillus Calmette-Guerin (BCG) therapy.

**Table 1:** Patient and disease characteristics of the cohorts

	Monopolar		Bipolar		*P* value
	N	%/IQR	N	%/IQR	
Number of patients, %	46	47.4%	51	52.6%	
Median age, IQR	75	69.5–84.5	73.5	64–83	0.82
Gender, %					0.45
Male	33	71.7%	40	78.4%	
Female	13	28.3%	11	21.6%	
Single tumour, %	25	54.3%	29	56.9%	0.81
Median tumour size (mm), IQR	20	10–30	15	7.5–22.5	0.75
CIS, %	4	8.7%	3	5.9%	0.59
Tumour stage, %					0.10
Ta	27	58.7%	38	74.5%	
T1	19	41.3%	13	25.5%	
Tumour grade, %					0.31
G1	5	13.9%	11	21.6%	
G2	23	50.0%	22	43.1%	
G3	18	39.1%	18	35.3%	

IQR = interquartile range; CIS = carcinoma-in-situ

Regarding the primary outcome, the RFS at one year were 59% in the monopolar arm and 69% in the bipolar arm. By 2-years, the RFS were 43% in the monopolar arm and 53% in the bipolar arm. The 5-year RFS were 37% in the monopolar arm and 47% in the bipolar arm. Overall, there was no significant difference in RFS (HR = 0.731; 95%CI = 0.433–1.236; *P* = 0.242) (Fig. 1a) between the two groups.

Other oncological outcomes including the PFS (HR = 1.014; 95%CI = 0.511–2.012; *P* = 0.969), CSS (HR = 0.718; 95%CI = 0.219–2.352; *P* = 0.584) and OS (HR = 1.135; 95%CI = 0.564–2.283; *P* = 0.722) were comparable between the two arms. Results were illustrated in the Kaplan-Meier survival analyses (Fig. 1b and d). Multivariate Cox regression analyses were performed to identify factors contributing to RFS and PFS. It was found that multifocal tumour (*p* < 0.001) was the only factors that was associated with worse RFS (Table 2) No significant factors were identified for PFS (Table 2b).

**Table 2:** Cox regression analysis on factors associated with survival outcomes

	Effect size	95% CI		*P* value
Recurrence-free survival				
Loop used (monopolar as reference)	0.6	0.345	1.044	0.07
Tumor size	1.012	0.994	1.03	0.18
Multifocality	3.347	1.872	5.984	**< 0.001**
Post-operative MMC	1.697	0.793	3.631	0.17
Tumour grade (G1 as reference)	0.977	0.629	1.52	0.92
Tumour stage (Ta as reference)	1.045	0.516	2.116	0.90
Presence of muscle in tumour base sampling	1.345	0.68	2.661	0.40
**Progression-free survival**				
Loop used (monopolar as reference)	0.783	0.449	1.368	0.73
Tumor size	1.002	0.984	1.021	0.49
Multifocality	2.205	1.235	3.935	0.07
Post-operative MMC	1.093	0.521	2.297	0.51
Tumour grade (G1 as reference)	1.157	0.731	1.83	0.16
Tumour stage (Ta as reference)	1.152	0.573	2.317	0.39
Presence of muscle in tumour base sampling	1.28	0.628	2.609	0.99

MMC = intravesical MMC treatment

Table 3 showed the periopertive outcomes. There were a total of 4 events (8.7%) noted in the monopolar and 4 in the bipolar group (7.8%). All the events were Clavien-Dindo grade I-II events. There were no severe complications reported. Of note, no incidence of bladder perforation was identified in the trial subjects.

**Table 3:** Peri-operative complications

	Monopolar		Bipolar		*P* value
	N	%	N	%	
Any grade complications	4	8.7%	4	7.8%	0.441
- Clavien Dindo I-II complications	4	8.7%	4	7.8%	
Haematuria	2	4.3%	3	5.9%	
Urinary retention	1	2.2%	1	2.0%	
Urinary tract infection	1	2.2%	0	0%	
- Clavien Dindo III-IV complications	0	0%	0	0%	

## Discussion

TURBT is the gold standard in the management of NMIBC. Whether bipolar TURBT is superior to monopolar TURBT and thus can totally replace it, remains an unanswered question. Existing randomised controlled trials that compared the two modalities focused on the ability of either of the methods in sampling muscle, or the presence of histological artefact (which are surrogates of resection quality) during operation. In the initial published analysis from the current RCT [10], it was demonstrated that bipolar TURBT (84.6%) was related to a better detrusor muscle sampling rate compared to monopolar TURBT (67.7%) in urothelial carcinoma (UC) cases. Del Rosso reported in their 132-patient randomised controlled trial that bipolar TURBT resulted in less histological artefact in the final specimen [13]. Whether such a difference translates to less recurrence and better progression-free survival is not fully accounted for.

Evidence that reported on the long-term oncological outcomes from the mode of TURBT was limited. In a post-hoc analysis of an international multicentre prospectively collected registry of 716 patients receiving TURBT, Liem and colleagues reported on the 12-month recurrence-free survival rates of monopolar TURBT at 70% and bipolar TURBT at 74% [14]. From a prospective study involving 240 patients receiving TURBT, Balci and colleagues reported the one-year bladder recurrence rate of monopolar TURBT at 4.3% with bipolar TURBT at 10.1%. Their result did not reach statistical significance [15]. Del Rosso and colleagues reported a similar conclusion from their randomised controlled trial of 113 patients. The 2-year recurrence-free survival rates were 60% for monopolar TURBT and 67% for bipolar TURBT in their cohort [13]. Results were inconclusive and follow-up was limited to a short to medium duration.

To the best of our knowledge, the current analysis is by far the first one that attempted to explore the long-term oncological outcomes of monopolar versus bipolar TURBT. It was noted that there was no significant difference in RFS and PFS between the two modalities. A seemingly better resection quality from bipolar TURBT does not directly translate to superior oncological outcomes. The postulation we are proposing is that NMIBC constitutes a wide spectrum of disease. Outcomes are thus governed by a host of factors. The EORTC studies enlightened us on the differences in prognosis between low and high-risk NMIBC [16]. In low-risk NMIBC, despite a marginal difference in resection quality, the probability of recurrence is not as nominal as the high-risk counterparts. And progression from low-risk disease is even rarer. Therefore, the modality of TURBT would be unlikely to make a substantial difference in survival data, within this group of less aggressive tumours. Whereas in high-risk cases, recurrence and progression rates were demonstrated to be higher, with 5-year recurrence reaching 80% (11). To minimise the risk of progression, much of it depends on the application of intravesical treatment, with intravesical Bacillus Calmette-Guerin being the major choice. As long as a complete resection is attained, the prognosis of this group of high-risk NMBIC is swayed by the subsequent treatment following TURBT. Despite the limited difference in the oncological efficacy demonstrated by the types of resection loops used, resection quality should still be a focus, in the attempt to reduce early tumour recurrence that is a direct result of tumour reseeding or even incomplete resection [17]. The methodology of resection appears to be more critical when it comes to preventing early recurrences, rather than the type of loops used. In recent years, the En Bloc TURBT (EBRT) had been proposed as a potentially superior alternative to TURBT [18] with fewer complications and better resection quality. The focus of future studies on effective transurethral resection would increasingly revolve around EBRT, and its comparative efficacy against conventional TURBT.

The merits of the current study should be highlighted. It is a prospective randomised trial with high study quality. The current study provided follow-up data of up to 5 years, while most previous studies have focused on short-term outcomes only. The extended analysis in this study allows for a more comprehensive comparative assessment of natural course of progression of this cohort. As we applied a rigorous inclusion and exclusion criteria, we were able to ensure a more homogeneous study population and focused specifically on the outcomes of NMIBC. Early follow-up protocol had been standardised and follow-up rates had been maximally safeguarded, to reduce cases lost to follow-up. On the other hand, the limitation of the current study would be its nature of being a post-hoc analysis. Constraints on retrieving outcome data were unavoidable. Another limitation was the lack of second look TURBT, as well as cystoscopy adjuncts (such as narrow band imaging or fluorescence cystoscopy), in the treatment protocol. Also, there is limitation in the documentation of subsequent intravesical treatment used. Moreover, as this study was conducted in a tertiary academic centre, whether our results would be generalisable to urology units of different calibres remains uncertain.

## Conclusion

Our study showed that monopolar TURBT is comparable to bipolar TURBT. Despite a higher detrusor muscle sampling rate in bipolar TURBT, there is no demonstrable difference between the two interventions in terms of long-term oncological outcomes.

## Electronic supplementary material

Below is the link to the electronic supplementary material.

Supplementary Material 1

Supplementary Material 2

## Data Availability

The authors agree to the sharing of the data involved in the current study, after written approval is attained from the Chinese University of Hong Kong.
