# Supplementary material for: Monopolar versus bipolar transurethral resection of bladder Tumour: post-hoc analysis of a prospective trial
Source: World J Urol. 2024 Aug 2;42(1):466. doi: 10.1007/s00345-024-05124-9 (PMC11297067; doi:10.1007/s00345-024-05124-9)
Supplement: Supplementary file 1 — Supplementary Material 1 [file 345_2024_5124_MOESM1_ESM.docx]

**Supplementary information (1)**

**Methods and materials**

*Study design*

This is a single centre, randomised phase III trial (RCT) conducted in a tertiary academic centre (8). We hereby report the long-term oncological outcomes following study intervention. Prior to the commencement of the study, ethics approval from the local authority had been granted. It was conducted in accordance with good clinical practice and the Declaration of Helsinki. It had been registered with ClinicalTrials.gov (NCT01581723).

*Patient selection*

All patients diagnosed with bladder tumour on diagnostic cystoscopy and planned for TURBT were screened for eligibility. Those aged above 18 who could provide informed consent were invited for recruitment. Patients who had undergone a previous TURBT within a 6-week period were excluded from recruitment.

*Randomization, allocation concealment, and blinding*

Informed consent was obtained at recruitment prior to the operation. Included patients were randomised to receive monopolar or bipolar TURBT. The treatment allocation ratio was 1:1. Research assistants utilised a computer-generated random number sequence for the randomisation results. The urologist responsible for the surgery would draw an envelope containing the information of the number sequence. Patients were blinded to the allocation. The pathologists responsible for specimen review were also blinded to the allocation.

*Treatment protocol*

Surgeries were performed under spinal or general anaesthesia. Cases were done by experienced surgeons who had performed more than 300 endourological cases and had previously performed both monopolar and bipolar resections. Monopolar TURBT was performed with an Olympus Monopolar HF-resection electrode (model A22205A). Settings were 90 W for cutting and 70 W for coagulation. The irrigant used during the procedure was 1.5% glycine solution. Bipolar TURBT was performed with an Olympus TURis 2.0 bipolar resection system with a bipolar HF-resection electrode (model WA22306D). Settings were 200 W for cutting and 120 W for coagulation. The irrigant during the procedure was 0.9% normal saline. Resections began from the exophytic portion of the lesion, followed by the stalk and the edges of the tumour. The muscle base was sampled separately. A single dose of intravesical mitomycin C was instilled within 24 hours following the surgery if there was no evidence of intraoperative bladder perforation and the tumour was resected completely.

*Study outcomes and post-hoc analysis*

The aim of the current study is to identify the long-term oncological outcomes of monopolar versus bipolar TURBT for NMIBC. In this analysis, only patients with final pathology of NMIBC included. Patients with benign pathology or muscle-invasive bladder cancer were excluded. Primary outcome was recurrence-free survival (RFS). Bladder cancer recurrence was based on endoscopic and histological diagnosis. Secondary outcomes included progression-free survival (PFS), cancer-specific survival (CSS) and overall survival (OS).

*Data collection and follow-up*

Baseline patient characteristics were obtained via the electronic documentation system. Data were collected prior to the admission for operation. Intraoperative details and perioperative outcomes were documented on a pre-set electronic form, with the aid of accessing patient charts. Patients would attend follow-up 4 weeks ± 2 weeks after the operation for pathology review and assessment of early postoperative outcomes. They had subsequent follow-up every 3 months since surgery until 12 months. Check cystoscopy was performed at every visit. Subsequent interval of check cystoscopy was based on EAU guidelines (9). Low risk cases would receive cystoscopy every year. For intermediate risk patients, subsequent cystoscopy would be arranged every 6 months. High risk patients would be receiving cystoscopy every 3months till two years post operation, then every 6 months for five years. Additional investigations such as computed tomography, positron emission tomography scan or magnetic resonance imaging would be arranged according to physician judgement.

*Sample size calculation*

The initial sample size was based on a calculation using detrusor muscle sampling rate as the outcome of interest (8). It was calculated with an 80% power at a significance level of 0.05, assuming a 65% and 85% detrusor muscle sampling rate for the monopolar and bipolar TURBT groups, respectively. Assuming a dropout rate of 10%, 160 patients was aimed for the study recruitment. In this study, only patients with final pathology of NMIBC were included, resulting in a total of 97 patients in the final analysis.

*Statistical analysis*

Statistical analyses were performed with SPSS version 24.0 (IBM Corporation, Armonk, NY, USA). Analysis was performed after applying the inclusion and exclusion criteria to the original cohorts. Categorical variables would be presented as percentages, and continuous variables would be presented as mean with standard deviation, or median with interquartile ranges. Independent sample t-test was used for parametric continuous variables and the chi-square test was used for categorical variables. A p-value of <0.05 was considered to be statistically significant. Kaplan-Meier survival plots would be adopted for the identification of the primary outcomes. Multivariate Cox regression analysis would be performed to identify contributing factors for RFS and PFS.
