# Supplementary figures and images for: Monopolar versus bipolar transurethral resection of bladder Tumour: post-hoc analysis of a prospective trial
Source: World J Urol. 2024 Aug 2;42(1):466. doi: 10.1007/s00345-024-05124-9 (PMC11297067; doi:10.1007/s00345-024-05124-9)

**Supplementary information (2)**

Consolidated Standards of Reporting Trials (CONSORT) diagram


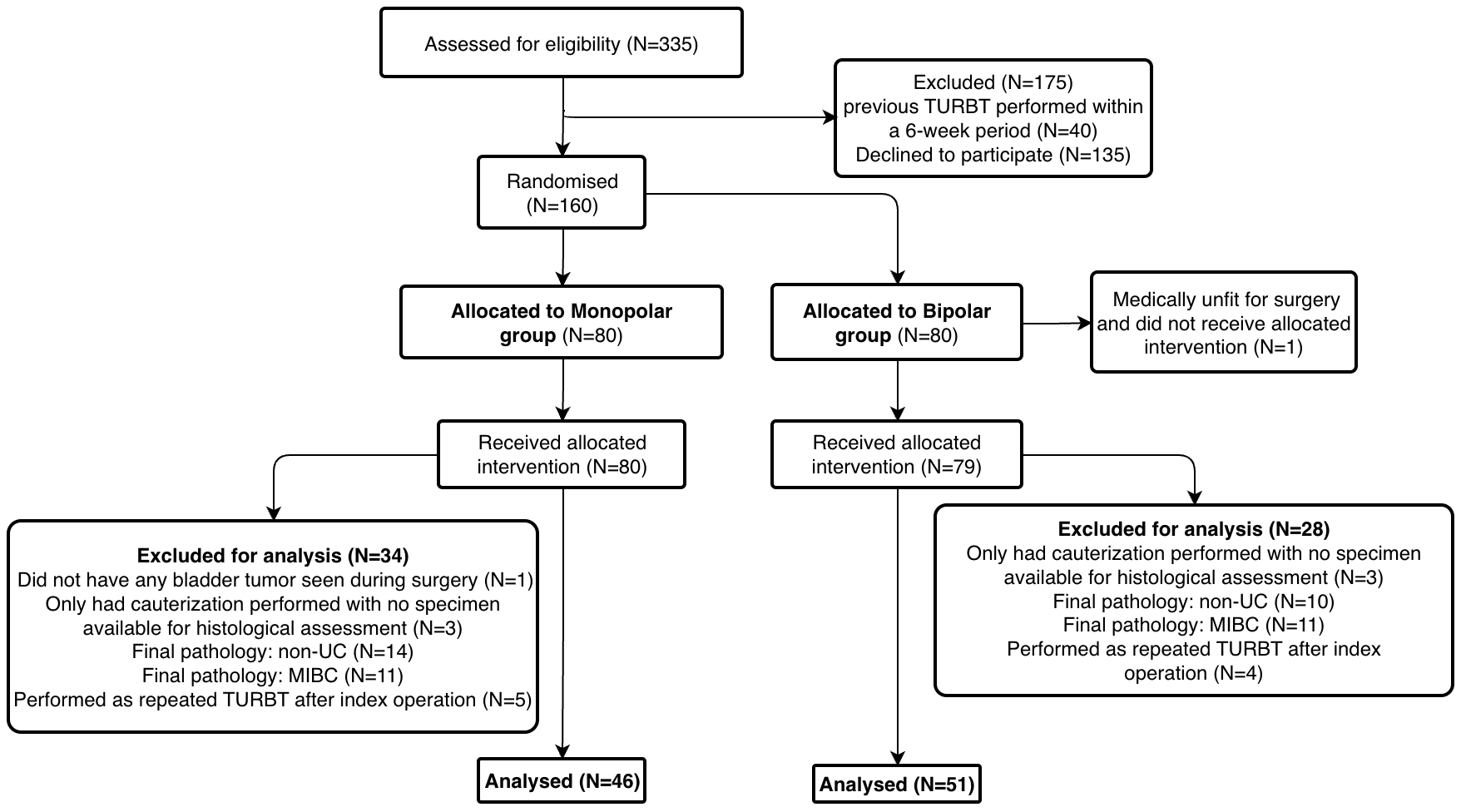

Supplement: Supplementary file 2 — Supplementary Material 2 [file 345_2024_5124_MOESM2_ESM.docx]
